# Episodic memory retrieval for story characters in high-functioning autism

**DOI:** 10.1186/2040-2392-4-20

**Published:** 2013-06-24

**Authors:** Hidetsugu Komeda, Hirotaka Kosaka, Daisuke N Saito, Keisuke Inohara, Toshio Munesue, Makoto Ishitobi, Makoto Sato, Hidehiko Okazawa

**Affiliations:** 1 The Hakubi Center for Advanced Research, Kyoto University, Kyoto, Japan; 2 Research Center for Child Mental Development, University of Fukui, Fukui, Japan; 3 Department of Neuropsychiatry, Faculty of Medical Sciences, University of Fukui, Fukui, Japan; 4 Biomedical Imaging Research Center, University of Fukui, Fukui, Japan; 5 Research Center for Child Mental Development, Kanazawa University, Kanazawa, Japan; 6 Department of Morphological and Physiological Sciences, Division of Cell Biology and Neuroscience, Faculty of Medical Sciences, University of Fukui, Fukui, Japan

**Keywords:** High-functioning autism, Narrative comprehension, Recognition, Memory retrieval, Similarity

## Abstract

**Background:**

The objective of this study was to examine differences in episodic memory retrieval between individuals with autism spectrum disorder (ASD) and typically developing (TD) individuals. Previous studies have shown that personality similarities between readers and characters facilitated reading comprehension. Highly extraverted participants read stories featuring extraverted protagonists more easily and judged the outcomes of such stories more rapidly than did less extraverted participants. Similarly, highly neurotic participants judged the outcomes of stories with neurotic protagonists more rapidly than did participants with low levels of neuroticism. However, the impact of the similarity effect on memory retrieval remains unclear. This study tested our ‘similarity hypothesis’, namely that memory retrieval is enhanced when readers with ASD and TD readers read stories featuring protagonists with ASD and with characteristics associated with TD individuals, respectively.

**Methods:**

Eighteen Japanese individuals (one female) with high-functioning ASD (aged 17 to 40 years) and 17 age- and intelligence quotient (IQ)-matched Japanese (one female) TD participants (aged 22 to 40 years) read 24 stories; 12 stories featured protagonists with ASD characteristics, and the other 12 featured TD protagonists. Participants read a single sentence at a time and pressed a spacebar to advance to the next sentence. After reading all 24 stories, they were asked to complete a recognition task about the target sentence in each story.

**Results:**

To investigate episodic memory in ASD, we analyzed encoding based on the reading times for and readability of the stories and retrieval processes based on the accuracy of and response times for sentence recognition. Although the results showed no differences between ASD and TD groups in encoding processes, they did reveal inter-group differences in memory retrieval. Although individuals with ASD demonstrated the same level of accuracy as did TD individuals, their patterns of memory retrieval differed with respect to response times.

**Conclusions:**

Individuals with ASD more effectively retrieved ASD-congruent than ASD-incongruent sentences, and TD individuals retrieved stories with TD more effectively than stories with ASD protagonists. Thus, similarity between reader and story character had different effects on memory retrieval in the ASD and TD groups.

## Background

Autism spectrum disorder (ASD) is diagnosed based on behavior such as difficulties with communication and social development, repetitive behavior, and narrowly focused but strong interests [1]. Difficulties with social interaction are particularly prominent in individuals with ASD [2,3]. Reciprocal social behavior refers to emotionally appropriate turn taking in social interactions with others. Although multiple sources of evidence show a lack of reciprocal social behavior in ASD [4], questions about whether individuals with ASD lack the capacity for reciprocal social behavior or whether they just fail to exhibit this behavior remain unanswered.

Stories are effective ways to examine the ability to engage in reciprocal social behavior, as they depict real life and include social interaction among story characters. Additionally, the ability to read between the lines is associated with the ability to follow the unwritten rules of social interaction that operate in daily life. In fact, stories have been used in many previous studies to investigate ‘theory of mind’ [5], which is the ability to infer the mental states of others, such as their intentions, beliefs, and desires. [6-8].

### Altered episodic memory in autism

Individuals with ASD follow organizational strategies during the retrieval of items from memory that differ from those of TD individuals [9,10]. Deep-level (semantic or episodic) processing of verbal materials enhances long-term memory better than does surface-level (phonological or perceptual) processing, a phenomenon known as the ‘levels-of-processing effect’. This assumes that retrieval is a function of trace elaboration at the time of encoding; that is, the deeper or more elaborate the encoding process is, the more likely it becomes that the information will be retrieved later [11]. Given that the self-related levels-of-processing effect was not found in individuals with ASD [12], it is likely that individuals with ASD use atypical retrieval systems.

Recognition memory has been used as an index of semantic memory, and source memory has been used as an index of episodic memory [13]. Compared with typically developing (TD) children, children with ASD show intact recognition but impaired source memory [14]. This difference in retrieval systems may be understood in terms of the well-established finding that individuals with ASD have impaired episodic memory [15]. Episodic memories are memories of personally experienced events that occurred in a particular place at a particular time. By contrast, semantic memories are memories of timeless, decontextualized facts and entail knowing about something outside the self rather than self-awareness [13]. Although individuals with ASD show deficits in episodic memory, they have intact semantic memory [16].

Some researchers have suggested that autobiographical memories, which are episodic memories about the self [17], have a narrative structure that includes characters, a temporo-spatial framework, causality, and goals [18-20]. Autobiographical memory may be impaired in ASD because individuals with ASD have difficulty integrating different event elements in the construction of consistent episodic memories [21].

### Similarity between readers and characters

According to a previous study [22], stories allow for the abstraction or generalization of complex social information in a form that offers personal enactments of experiences, rendering them more comprehensible than they would otherwise be. The abstraction function performed by fictional stories requires that readers place themselves into the story events [22]. This abstraction process should be facilitated when readers have characteristics similar to those of protagonists, as this would presumably enhance the retrieval of memories related to story events or characters.

We predicted that similarities between readers and characters in stories would facilitate the cognitive processing involved in story comprehension. This prediction, which is known as the ‘similarity hypothesis’ [23,24], is supported by several studies. Recent studies on TD adults have shown that similarities (or dissimilarities) between readers and characters play critical roles in reading stories [25]. For example, personality is an important factor contributing to interactions between readers and characters [23,24]. It is easier for highly extraverted than for less extraverted readers to understand stories about a highly extraverted character [23]. Additionally, highly extraverted readers can judge the behavioral outcomes of highly extraverted characters more rapidly than can less extraverted readers, and highly neurotic readers can judge the outcomes of stories with highly neurotic characters more rapidly than can less neurotic readers [24]. Although previous studies have shown that personality similarities between readers and characters facilitated reading comprehension, it remains unclear whether this facilitation effect extends to memory retrieval. Based on the similarity hypothesis, we predicted that memory retrieval would be enhanced when readers with ASD read stories featuring protagonists with characteristics of ASD and when readers with TD read stories with TD protagonists. The symmetry predicted by this hypothesis rests on the assumption that ASD and TD can be understood as existing on a spectrum such that the same processes would underpin perception in those with ASD and in TD individuals.

### Objectives of the current project

The objective of the current study was to examine differences between individuals with ASD and TD individuals with regard to episodic memory retrieval for story characters. Half the stories featured a protagonist with characteristics of ASD, and half featured a protagonist with non-autistic characteristics usually observed in TD individuals. We assumed that readers and characters were similar when individuals with ASD read stories featuring protagonists with characteristics of ASD and when TD individuals read stories featuring protagonists with the characteristics of TD individuals. If similarity between readers and characters facilitates memory retrieval, recognition times should be shorter when such similarity exists. If similarity between readers and characters does not influence memory representation, no differences between the groups should be observed in this regard.

## Methods

### Participants

Eighteen Japanese individuals (one female) with high-functioning ASD (aged 17 to 40 years) were recruited by the Department of Neuropsychiatry of the University of Fukui Hospital, Japan, and the Department of Psychiatry and Neurobiology of Kanazawa University Hospital, Japan. The second and fifth authors diagnosed the participants based on the Diagnostic and Statistical Manual of Mental Disorders (DSM-IV-TR) [1] and standardized criteria taken from the Diagnostic Interview for Social and Communication Disorders (DISCO) [26]. The DISCO is reported to have good psychometric properties [27]. It also contains items addressing early development and a section focused on activities of daily living, which provides data on the individual’s level of functioning in several areas in addition to the social and communication domains [26]. The ASD group consisted of 13 participants with autistic disorder and five with Asperger’s disorder. Seventeen age- and intelligence quotient (IQ)-matched Japanese (one female) TD participants (aged 22 to 40 years) were recruited from the local community (Table 1). Participants were excluded if they had a history of major medical or neurological illness, including epilepsy, significant head trauma, or alcohol or drug dependence. They were screened to exclude individuals who had a first-degree relative with an axis I disorder according to DSM-IV criteria. IQ assessments were performed with the Wechsler Adult Intelligence Scale-III (WAIS-III) [28]. All participants had full-scale IQ scores > 80. The groups did not differ in terms of age, verbal IQ, or full-scale IQ (Table 1). To quantify participants’ autistic traits, we used the autism- spectrum quotient (AQ) [29], which consists of subscales measuring communication, social skills, attention switching, imagination, and attention to detail. Although AQ scores (mean ± standard deviation (*SD*) = 31.5 ± 8.0 for the ASD group and 14.6 ± 5.8 for the TD group) are not diagnostic, this measure provides useful supportive diagnostic information, as it has been validated in a clinical sample [30]. The protocol of this study was approved by the ethics committee of the University of Fukui. After a complete explanation of the study, all participants provided written, informed consent prior to participation.

**Table 1:** Mean ages and IQ scores of the participants

	**ASD group**	**TD group**	***t***	***p***
	**(*n*= 18)**	**(*n*= 17)**		
Age in years	26.3 (6.7)	26.9 (5.3)	-.30	.77
Full Scale IQ	105.3 (14.1)	110.4 (7.0)	−1.3	.20
*(Range)*	*(81 to 133)*	*(96 to 126)*		
Verbal IQ	111.1 (16.6)	112.9 (8.1)	-.41	.69
*(Range)*	*(84 to 147)*	*(100 to128)*		
Performance IQ	95.8 (14.9)	105.4 (7.1)	−2.4	.02
*(Range)*	*(75 to 124)*	*(92 to 120)*		
Total AQ	31.5 (8.0)	14.6 (5.8)	7.1	.00
*(Range)*	*(18 to 48)*	*(7 to 26)*		
Communication	6.1 (3.2)	2.1 (2.4)	4.2	.00
*(Range)*	*(0 to 10)*	*(0 to 7)*		
Social skills	6.8 (2.4)	3.1 (2.4)	4.1	.00
*(Range)*	*(1 to 10)*	*(0 to 9)*		
Attention switching	7.4 (1.7)	3.6 (2.0)	6.1	.00
*(Range)*	*(5 to 10)*	*(0 to 8)*		
Imagination	5.1 (1.8)	2.8 (1.6)	3.9	.00
*(Range)*	*(2 to 8)*	*(0 to 6)*		
Attention to detail	6.2 (2.6)	3.1 (1.7)	4.1	.00
*(Range)*	*(1 to 10)*	*(0 to 7)*		

Means (*SD*s) are presented. Range of scores on each measure is shown by italic fonts.

### Reading task

We wrote stories featuring characters with and without autistic characteristics. The adult version of the social responsiveness scale (SRS)^a^ was used to describe the autistic characteristics mentioned in the stories [31,32]. The SRS, which has child and adult versions, is a quantitative measure of autistic traits and covers a continuous range from significantly impaired to above average [2,3,33,34]. Consistent with previous evidence of the significant heritability of the broader autism phenotype [35-37], several previous studies have reported significant familial correlations for the subclinical features of ASD measured by the SRS [2].

**Figure 1:**
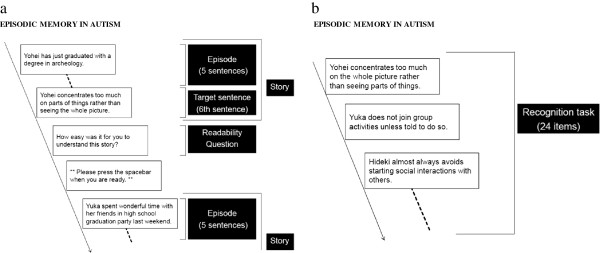
**Procedures of the experiment. a)** Procedures of reading tasks. **b**) Procedures of recognition tasks.

We constructed 24 stories; 12 stories featured protagonists with characteristics of ASD, and the other 12 featured protagonists with characteristics of TD individuals. Each story contained five-sentence episodes (story setting and description of the protagonist’s characteristics) and a target (outcome) sentence (Tables 2 and 3, Figure 1a). Half the stories had male protagonists, and half had female protagonists. Protagonists in the ASD episodes possessed autistic characteristics drawn from the Japanese version of the SRS [38,39]. For example, Table 2 presents Yohei (Japanese male name) as experiencing difficulty perceiving wholes, which was based on the SRS item ‘concentrates too much on parts of things rather than seeing the whole picture’. Protagonists in the TD episodes did not exhibit characteristics of ASD. For example, Yuka (Japanese female name) enjoyed joining group activities (Table 3). This episode was constructed to depict a trait opposite to the SRS item ‘does not join group activities unless told to do so’.

**Table 2:** Sample story with ASD episode

**ASD episode**	
Yohei has just graduated with a degree in archeology.	
As a graduation gift, his uncle sent him on an expedition to the Yucatan in Mexico.	
While there, Yohei went into the jungle to examine the ancient Mayan pyramids.	
In front of the pyramids, he noticed a rock that looked strangely like a ‘daruma’ (roly-poly papier-mache doll).	
Although his uncle said to him, ‘why don’t you examine pyramids?’ he was immersed in the rock in front of him and was taking pictures of it with absorbed interest.	
Congruent with ASD episode	Incongruent with ASD episode
Yohei concentrates too much on parts of things rather than seeing the whole picture.	Yohei concentrates too much on the whole picture rather than seeing parts of things.

**Table 3:** Sample story with TD episode

**TD episode**	
Yuka spent a wonderful time with her friends at a high school graduation party last weekend.	
She made a plan to travel with new friends from her university.	
She also looked for new members for the university tennis circle she will want to join.	
She attended a commencement today, and she graduated top of the class.	
Yuka asked her friend to help select her dress for the graduation ceremony.	
Congruent with TD episode	Incongruent with TD episode
Yuka joins group activities without being told to do so.	Yuka does not join group activities unless told to do so.

ASD episodes that had an ASD target sentence were considered congruent (Tables 2 and 3). Thus, the participants read a congruent or an incongruent outcome for each episode. Participants did not read the same episodes or target sentences twice, and presentation of stories was randomized.

### Recognition task

After reading the 24 stories, participants were asked to complete a recognition task about the target sentence (ASD or TD outcome sentence) of each story (Figure 1b). They were instructed to decide whether the target sentence had appeared in the stories they read. For example, participants who read, ‘Yohei concentrates too much on parts of things rather than seeing the whole picture’ were later asked to judge whether the sentence, ‘Yohei concentrates too much on the whole picture rather than seeing parts of things’ was OLD (the participants had read the sentence) or NEW (the participants had not read the sentence).

This recognition task enabled us to examine congruence effects with different sources (episodes). Four types of sentences were used: those congruent with ASD episodes, those congruent with TD episodes, those incongruent with ASD episodes, and those incongruent with TD episodes. Thus, the effects of group (ASD or TD), congruence (congruent or incongruent with the episode), and episode (ASD or TD) as well as the interaction among group, congruence, and episode were examined.

### Procedure

Figures 1a and b show the flow of the present experiment. Stories were presented one sentence at a time on a computer (Figure 1a). Participants read a single sentence and pressed a spacebar to advance to the next sentence; inter-stimulus interval (ISI) = 0. After reading the final (sixth) sentence of each story, we presented the following question: ‘how easy was it for you to understand this story?’ Participants rated readability on a seven-point scale (1: very easy, 4: neither easy nor difficult, 7: very difficult). After reading all 24 stories, they were asked to complete a recognition task about the final sentence of each story (Figure 1b). Participants were not told about this recognition test before reading the stories. The experiment lasted approximately 30 minutes.

## Results

### Memory encoding

We collected the reading times for target sentences (Additional file 1). Reading times that were more than 2.5 SDs above the mean for each participant were eliminated because it has been recommended that SD cutoffs be used to analyze response-time data as they reflect the variability of subject means [40].

We conducted a three-way analysis of variance (ANOVA) on reading time with group as a between-participant factor (ASD versus TD) and congruence (congruent versus incongruent) and episode (ASD versus TD) as within-participant factors^b^. The main effects of congruence and story episode were significant (*F*(1, 33) = 38.00, *p* < .05, *MS*_*e*_ = 53723589.61, *P*rep = .99, η_p_^2^ = .54; *F*(1, 33) = 22.43, *p* < .05, *MS*_*e*_ =14408486.00, *P*rep = .99, η_p_^2^ = .40). Thus, the reading times for target sentences that were incongruent with episodes were longer than those for sentences congruent with episodes, and the reading times for ASD episodes were longer than those for TD episodes.

We compared the ASD and TD groups based on the readability ratings of the stories (Additional file 2). We conducted a three-way ANOVA of readability ratings with group as a between-participant factor (ASD versus TD) and congruence (congruent versus incongruent) and episode (ASD versus TD) as within-participant factors. The three-way interaction (*F*(1, 33) = 0.03, *p* > .05), the two-way interaction between group and congruence (*F*(1, 33) = 0.00, *p* > .05), and the two-way interaction between group and episode (*F*(1, 33) = 0.88, *p* > .05) were not significant. However, the two-way interaction between congruence and episode was significant (*F*(1, 33) = 13.64, *p* < .05, *MS*_*e*_ = 6.26, *P*rep = .99, η_p_^2^ = .29). The simple interaction effects showed that incongruent stories were more difficult than were congruent stories irrespective of whether they were embedded in ASD or TD episodes (*p* < .05).

### Memory retrieval

The mean accuracy rates for the ASD group were 72.45% (*SD* = 18.80)^c,^ and those for the TD group were 71.32% (*SD* = 18.18)^d.^ We observed no significant three-way interactions (*F*(1, 33) = 1.37, *p* > .05), no interaction between group and congruence (*F*(1, 33) = 0.10, *p* > .05), no interaction between group and episode (*F*(1, 33) = 0.12, *p* > .05), and no interaction between congruence and episode (*F*(1, 33) = 1.37, *p* > .05).

**Figure 2:**
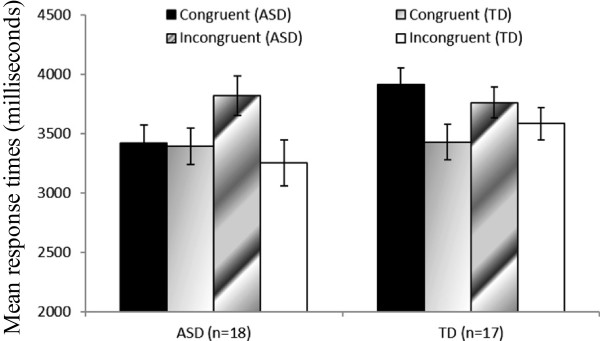
**Differences in response times in the recognition task in the ASD and the TD groups.** Error bars represent the standard errors.

**Figure 3:**
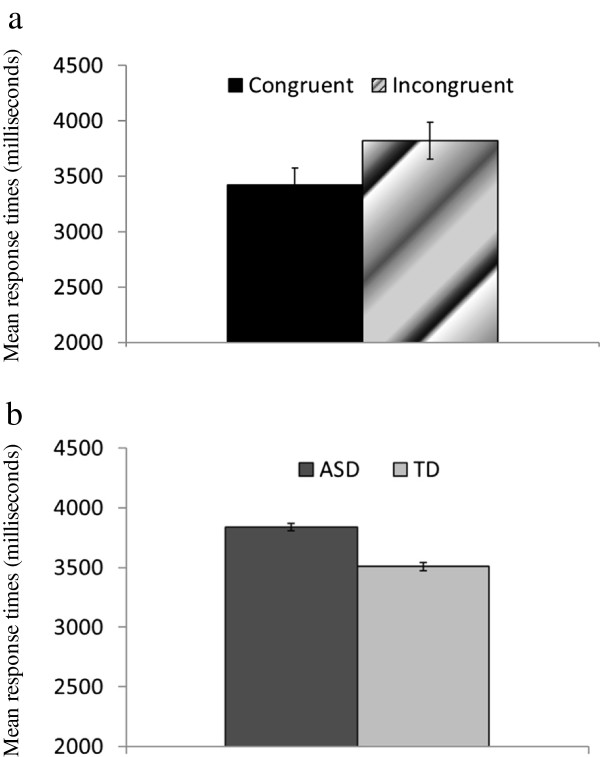
**Differences of response times for memory retrieval. a**) Differences in memory retrieval times between target sentences that were congruent versus incongruent with the ASD episodes in the ASD group. Error bars represent the standard errors. **b**) Differences in memory retrieval times between ASD and TD stories in the TD group. Error bars represent the standard errors.

We compared the ASD and TD groups based on the response times in the recognition task (Figure 2). The response times for correct responses on the recognition task were analyzed under the assumption that the time taken to respond to items reflects retrieval difficulties; specifically, shorter response times suggest easier retrieval in recognition tasks [41,42]. Response times more than 2.5 SDs above the mean for each participant were eliminated. We conducted a three-way ANOVA on response times with group as a between-participant factor and congruence (congruent versus incongruent) and episode (ASD versus TD) as within-participant factors. The three-way interaction was significant (*F*(1, 33) = 4.41, *p* < .05, *MS*_*e*_ = 357662.55, *P*rep = .92, η_p_^2^ = .12). The patterns of simple effects differed between the ASD and TD groups. The ASD group retrieved sentences congruent with ASD episodes faster than they retrieved sentences incongruent with ASD episodes Figure 3a; (*F*(1, 17) = 4.80, *p* < .05, *MS*_*e*_ = 301033.89, *P*rep = .92, η_p_^2^ = .22), and the ASD group retrieved sentences incongruent with ASD episodes more slowly than they retrieved sentences incongruent with TD episodes (*F*(1, 17) = 9.90, *p* < .05, *MS*_*e*_ = 291818.54, *P*rep = .97, η_p_^2^ = .37). Furthermore, the TD group retrieved TD stories faster than they retrieved ASD stories (Figure 3b; *F*(1, 16) = 4.63, *p* < .05, *MS*_*e*_ = 403699.27, *P*rep = .92, η_p_^2^ = .22).

### Correlation between AQ scores and psychological measures

We calculated correlations between AQ scores and the reading times for target sentences to investigate the relationship between reading processes and the characteristics of ASD. Table 4 shows that total AQ scores and scores on the communication and social skills subscales were significantly correlated with the reading times for TD episodes (congruent and incongruent sentences embedded in TD episodes) in the Total groups. Attention-to-detail scores were significantly correlated with reading times for congruent sentences embedded in TD episodes.

**Table 4:** Correlations between AQ and target sentence reading times

	**Total (*N*= 35)**				**ASD (*n*= 18)**				**TD (*n*= 17)**			
**Congruencies**	**Congruent**		**Incongruent**		**Congruent**		**Incongruent**		**Congruent**		**Incongruent**	
Episode	ASD	TD	ASD	TD	ASD	TD	ASD	TD	ASD	TD	ASD	TD
Total AQ	.32	.43^a^	.31	.39^a^	.26	.64^a^	.50^a^	.43	.18	.18	. 02	.39
Communication	.31	.39^a^	.30	.40^a^	.31	.51^a^	.44	.44	.02	.12	-.05	.23
Social skills	.27	.40^a^	.24	.42^a^	.19	.34	.37	.37	.16	.45	.02	.46
Attention switching	.26	.24	.17	.25	.15	.45	.26	.25	.14	-.15	-.05	.11
Imagination	.16	.24	.27	.31	.00	.32	.29	.24	.12	-.02	.19	.29
Attention to detail	.23	.35^a^	.24	.13	.13	.52^a^	.29	.10	.06	-.12	-.01	-.14

^a^*p* < .05, two-tailed.

In the ASD group, total AQ scores were significantly correlated with the reading times for congruent sentences embedded in TD episodes and with the reading times for incongruent sentences embedded in ASD episodes. Scores for communication and attention to detail were significantly correlated with the reading times for congruent sentences embedded in TD episodes.

We found no significant correlations between total AQ scores and other psychological measures, readability, or response time or accuracy in recognition tasks. Thus, participants’ AQ scores had an impact on encoding (reading times) but not on retrieval processes.

## Discussion

Only the correlation analyses revealed differences between the ASD and TD groups in encoding processes. AQ scores were significantly correlated with reading speed, and memory retrieval processes were different between the groups. Although the memory of individuals with ASD was as accurate as that of TD individuals, differences in response times reflected different patterns of memory retrieval.

### Similarity hypothesis

The similarity hypothesis [23,24] predicts that cognitive processes are enhanced when one is focused on similar others. In the current study, this hypothesis predicts that the response times for stories featuring protagonists similar to the participants should be faster than those for stories featuring dissimilar protagonists. Although individuals with ASD did not differ from TD individuals in story-encoding processes such as reading times and readability measures, they did differ in the retrieval of sentences from memory. The ASD group retrieved ASD-congruent sentences faster than they retrieved ASD-incongruent sentences (Figure 3a), and the TD group retrieved TD stories faster than they retrieved ASD stories (Figure 3b). Thus, the similarity hypothesis was supported in the TD group.

Although the ASD group did not retrieve ASD stories faster than they retrieved TD stories, they showed specific patterns of responses to protagonists with ASD characteristics. That is, the ASD group may have detected incongruence in the protagonists in ASD episodes based on their own prior experiences.

### Memory encoding processes

When readers read an outcome sentence that is incongruent with the prior context, they tend to resolve the incongruence by inferring the meaning of the sentence using their own knowledge of the world [43]. These efforts involve an additional cognitive load and increase the time spent reading the outcome sentence [44]. In the current study, we found no significant interaction between group membership and the main character’s characteristics in terms of reading times. These results showed that regardless of the protagonist’s characteristics (ASD or TD), individuals with ASD detected incongruence as well as TD individuals did.

The readability results also did not show significant group differences. ASD and TD groups both found it more difficult to understand incongruent than congruent stories [45,46]. Additionally, we observed no significant interactions between group and protagonist characteristics in the readability ratings. These results suggest that individuals with ASD are able to understand and monitor the consistency of stories regardless of the characteristics of the protagonist. Thus, our similarity hypothesis was not supported with respect to encoding processes, as measured by reading time and readability measures.

Correlation analyses revealed that total AQ scores and difficulties involving communication and social skills were associated with longer reading times for stories with TD episodes in the Total group. These results suggest that it takes more time for readers with social problems affecting communication and social skills to read stories whose protagonists had characteristics of TD individuals. In the ASD group, total AQ scores were associated with longer reading times for congruent sentences embedded in TD episodes and incongruent sentences embedded in ASD episodes. Thus, individuals with ASD who have high AQ scores have difficulties integrating TD target sentences with the previous context during memory encoding processes.^e^

On the other hand, attention to detail, which is a non-social feature measured by the AQ [47], was associated with longer reading times for congruent sentences embedded in TD episodes. This suggests that the tendency to attend to details is related to understanding congruent stories featuring TD characters. As individuals with ASD are characterized by their attention to detail [47], they are likely to attend to the differences between themselves and TD individuals when they read stories with TD characters.

Given that the correlation between reading times and AQ scores was not significant in the TD group, the significant overall correlation for all respondents must be attributable to the ASD group. Thus, these results reflect variation within the ASD group, suggesting heterogeneity in ASD [48].

### Memory retrieval processes

Data on reading times revealed no significant interactions between group and target sentences or between group and episode. Thus, processing at the level of encoding did not explain the differences in the retrieval processes for ASD and TD target sentences in the story-reading task. Instead, differences in retrieval strategies led to differences between ASD and TD groups in the recognition task.

Individuals with ASD did not exhibit deficits in recognition accuracy. Thus, the differences between ASD and TD individuals in the cognitive processes underlying memory were reflected in response times during memory retrieval. These results are consistent with the notion that children with ASD have intact recognition but impaired source memory [14]. Because individuals with ASD did not show deficits in recognition, they probably relied on strategies based on altered source memory. The differences in the ‘levels-of-processing effect’ between the ASD and TD groups may explain these strategic differences. The ASD group engaged in a deeper level of processing in response to ASD stories, which gave them an advantage in detecting the incongruences in these stories. Their processing of characters similar to themselves was facilitated by their ability to abstract, which made the story more comprehensible than it would ordinarily be [22]. Although previous studies have suggested that individuals with ASD do not exhibit a self-related levels-of-processing effect [12], the present study showed a levels-of-processing effect for story characters with ASD characteristics, which is related to the experience of self in individuals with ASD.

### Future directions

Three directions should be considered for future research. Firstly, the autobiographical memory of individuals with ASD should be examined. In 2010, Lind distinguished between autobiographical episodic memory and non-autobiographical episodic memory. Individuals with ASD show impaired autobiographical episodic memory and a reduced self-reference effect (which may rely on psychological aspects of the self-concept) but do not show specific impairments in memory for their own rather than others’ actions (which may rely on physical aspects of the self-concept) [11]. Although our study found altered episodic memory retrieval among individuals with ASD, the role of self-concept in episodic memory retrieval remains unclear. The autobiographical and non-autobiographical episodic memories of individuals with ASD are worth examining.

Secondly, we need to investigate the degree to which similarity mediates memory retrieval and reading processes in story comprehension. Previous studies [23] examined people with different personality traits to manipulate similarities between readers and characters (for example, highly extraverted readers should be similar to extraverted story characters). Future studies should further examine interactions between individuals with and without ASD and personality traits.

Thirdly, it is important to examine the effect of similarity on reading comprehension. Similarities between readers’ and characters’ personality traits elicit empathetic responses [24]. Readers’ preferences and the amount of empathy they feel toward similar characters should be investigated to understand readers’ involvement in fictional stories [49-51]. Individuals with ASD likely prefer familiar situations and empathize with others with autistic characteristics [52].

## Conclusions

Individuals with ASD were more effective in retrieving ASD-congruent than ASD-incongruent sentences, and TD individuals were more effective at retrieving TD than ASD stories. Thus, the similarity between readers and story characters has a different impact on memory retrieval in ASD and TD groups.

The examination of the reading and memory strategies followed by ASD individuals is essential for developing effective interventions in medical and educational settings [53-55]. As these findings help explain the characteristics of individuals with ASD, they may also contribute to improving special needs education, educational interventions, and literacy education programs for these individuals.

## Endnotes

^a^Use of the Japanese version of the SRS was permitted by Western Psychological Services.

^b^The three-way interaction (*F*(1, 33) = 1.35, *p* > .05), the two-way interaction between group and congruence (*F*(1, 33) = 0.05, *p* > .05), the two-way interaction between group and episode (*F*(1, 33) = 0.07, *p* > .05), and the two-way interaction between congruence and episode (*F*(1, 33) = 1.94, *p* > .05) were not significant.

^c^Accuracy rates were 72.22% (*SD* = 20.61) for sentences congruent with ASD episodes, 71.30% (*SD* = 21.12) for those congruent with TD episodes, 68.52% (*SD* = 16.06) for those incongruent with ASD episodes, and 77.78% (*SD* = 17.15) for those incongruent with TD episodes.

^d^Accuracy rates were 67.65% (*SD* = 19.96) for sentences congruent with ASD episodes, 75.49% (*SD* = 16.79) for those congruent with TD episodes, 68.63% (*SD* = 18.52) for those incongruent with ASD episodes, and 73.53% (*SD* = 17.74) for those incongruent with TD episodes.

^e^Because no significant correlations between total AQ scores and recognition measures were observed, AQ scores explained difficulties in memory encoding but not in memory retrieval processes.

## Abbreviations

AQ: Autism-spectrum quotient; ASD: Autism spectrum disorders; IQ: Intelligence quotient; ISI: Inter-stimulus interval; TD: Typically developing.

## Competing interests

The authors declare that they have no competing interests.

## Authors’ contributions

HK (first author) was involved in conceiving, designing, and conducting the experiment, analyzing and interpreting data, and drafting the article. HK (second author) was involved in conceiving and conducting the experiment, interpreting the data, and drafting the article. DNS and TM were involved in conducting the experiment and interpreting the data. KI, MI, MS, and HO were involved in interpreting the data. All authors read and approved the final manuscript.

## Supplementary Material

Additional file 1 **Mean reading times of target sentences in milliseconds by the ASD and the TD group.** Error bars represent the standard errors. There were no significant interactions. There were significant main effects of congruencies and episodes (*p*s < .05). Congruent < incongruent (*p* < .05). TD episodes < ASD episodes (*p* < .05). Click here for file

Additional file 2 **Mean values of readability for the ASD and the TD groups.** Error bars represent the standard errors. The question was ‘how easy was it for you to understand this story’? (Seven-point scale: 1: very easy, 4: neither easy nor difficult, 7: very difficult). There were no significant group differences. There was a significant interaction between congruence and story episodes (*p* < .05). Congruent with ASD episodes < incongruent with ASD episodes (*p* < .05). Congruent with TD episodes < incongruent with TD episodes (*p* < .05). Congruent with ASD episodes > congruent with TD episodes (*p* < .05). Click here for file
