# Supplementary material for: Episodic memory retrieval for story characters in high-functioning autism
Source: Mol Autism. 2013 Jun 24;4:20. doi: 10.1186/2040-2392-4-20 (PMC3695882; doi:10.1186/2040-2392-4-20)
Supplement: Additional file 1 — Mean reading times of target sentences in milliseconds by the ASD and the TD group. Error bars represent the standard errors. There were no significant interactions. There were significant main effects of congruencies and episodes (ps < .05). Congruent < incongruent (p < .05). TD episodes < ASD episodes (p < .05). [file 2040-2392-4-20-S1.doc]

#
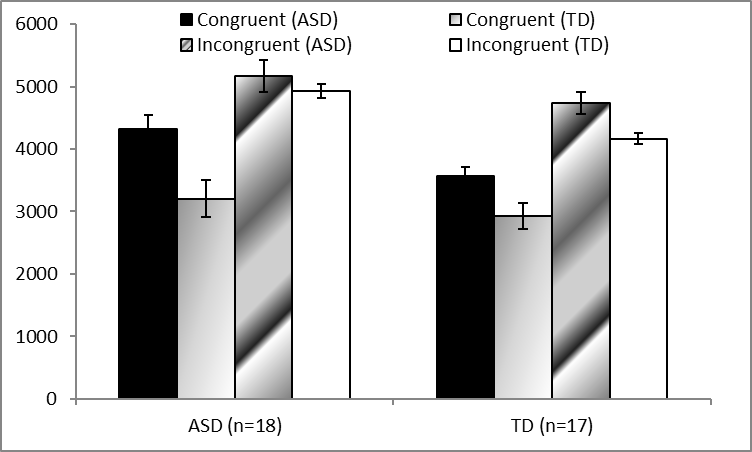


Additional file 1. Mean reading times of target sentences in milliseconds (SDs) by the ASD and the TD group.

Mean reading times (milliseconds)

Error bars represent the standard errors.

There were no significant interactions. There were significant main effects of congruencies and episodes (*p*s < .05).

Congruent < Incongruent (*p* < .05).

TD episodes < ASD episodes (*p* < .05).
