# Supplementary material for: Episodic memory retrieval for story characters in high-functioning autism
Source: Mol Autism. 2013 Jun 24;4:20. doi: 10.1186/2040-2392-4-20 (PMC3695882; doi:10.1186/2040-2392-4-20)
Supplement: Additional file 2 — Mean values of readability for the ASD and the TD groups. Error bars represent the standard errors. The question was ‘how easy was it for you to understand this story’? (Seven-point scale: 1: very easy, 4: neither easy nor difficult, 7: very difficult). There were no significant group differences. There was a significant interaction between congruence and story episodes (p < .05). Congruent with ASD episodes < incongruent with ASD episodes (p < .05). Congruent with TD episodes < incongruent with TD episodes (p < .05). Congruent with ASD episodes > congruent with TD episodes (p < .05). [file 2040-2392-4-20-S2.doc]

#
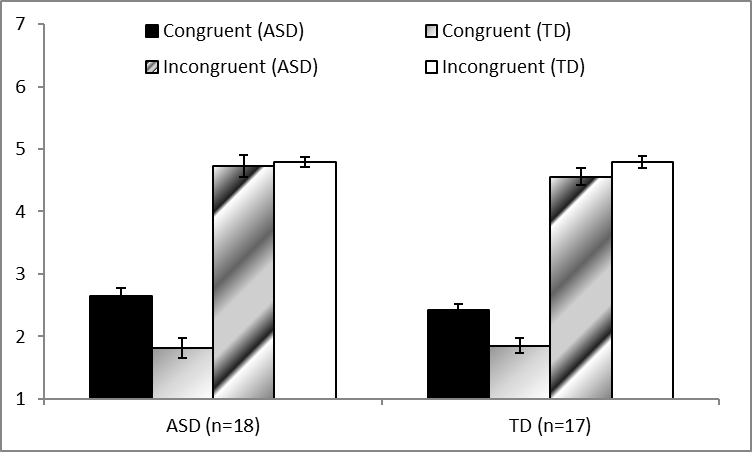


Mean rating values

# Additional file 2 – Mean values of readability for the ASD and the TD groups.

Error bars represent standard errors.

The question was “How easy was it for you to understand this story?” (Seven-point scale: 1: very easy, 4: Neither easy nor difficult, 7: very difficult).

There were no significant group differences. There was a significant interaction between congruence and story episodes (*p* < .05).

Congruent with ASD episodes < incongruent with ASD episodes (*p* < .05).

Congruent with TD episodes < incongruent with TD episodes (*p* < .05).

Congruent with ASD episodes > congruent with TD episodes (*p* < .05).
